# Community Participation in Two Vaccination Trials in Slums of Kolkata, India: A Multi-level Analysis

**DOI:** 10.3329/jhpn.v28i5.6153

**Published:** 2010-10

**Authors:** Mohammad Ali, Dipika Sur, Anna Lena Lopez, Suman Kanungo, R. Leon Ochiai, Byomkesh Manna, Deok Ryun Kim, Jacqueline Deen, Sujit K. Bhattacharya, John D. Clemens

**Affiliations:** ^1^ International Vaccine Institute, Seoul, Korea; ^2^ National Institute of Cholera & Enteric Diseases, P-33 CIT Road, Scheme XM, Kolkata 700 010, India; ^3^ Indian Council of Medical Research, New Delhi 110 029, India

**Keywords:** Cholera, Cholera vaccines, Typhoid fever, Typhoid-paratyphoid vaccines, Vaccination, India

## Abstract

This study aims at understanding the individual and community-level characteristics that influenced participation in two consecutive vaccine trials (typhoid and cholera) in urban slums of Kolkata, India. The study area was divided into 80 geographic clusters (communities), with 59,533 subjects aged ≥2 years for analysis. A multi-level model was employed in which the individuals were seen nested within the cluster. Rates of participation in both the trials were nearly the same; those who participated in the initial trial were likely to participate in the subsequent cholera vaccine trial. Communities with predominantly Hindu population, lower percentage of households with an educated household head, or lower percentage of households owning a motorbike had higher participation than their counterparts. At individual scale, higher participation was observed among younger subjects, females, and individuals from households with a household head who had no or minimal education. Geographic patterns were also observed in participation in the trials. The results illustrated that participation in the trial was mostly influenced by various individual and community-level factors, which need to be addressed for a successful vaccination campaign.

## INTRODUCTION

Immunization is one of the most cost-effective strategies to prevent millions of infectious disease episodes and deaths around the world. Phase III and IV vaccine trials are crucial in the licensure of newly-developed vaccines or deployment of underused vaccines in poor countries. Phase III randomized controlled trials enroll thousands of subjects to provide rigorous evidence about vaccine protection against naturally-occurring infections. Results of phase IV studies, which may be even larger in size and longer in duration of follow-up, are used for evaluating the safety and effectiveness of licensed vaccines when given under public-health conditions. The large numbers of participants, prospective nature of the studies, lengthy duration of follow-up, quality control, and quality-assurance procedures make these trials time-, labour- and cost-intensive.

The success of large phase III and IV trials and mass-vaccination campaigns in developing-country populations requires information dissemination, discussions with community leaders, and encouragement of community participation. Despite these efforts, not all members of a population may wish to participate in the vaccine trial or the campaign. Each individual and family need to weigh perceived risks and benefits, reflect on the value of participation, and consider potential consequences (1). In some developing-country societies, false perceptions and irrational fears about vaccines may appear when conducting field trials and mass-vaccination campaigns. Such a situation occurred in India during the 1970s when vaccines were associated with fears of family-planning agenda (2,3). In these types of situation, a vaccine trial or a campaign may become vulnerable to misperceptions and fears, which can greatly influence community participation.

Typhoid fever and cholera are endemic in India and are persistent problems in Kolkata (4). India, along with some Asian countries, is considered a high typhoid-incidence area, with a crude incidence of 622 cases per 100,000 people per year (5). The typhoid Vi polysaccharide vaccine is licensed in India but its cost precludes it from being used in the public-health setting. Cholera has a tenacious grip on the state of West Bengal where it is endemic. There was no licensed cholera vaccine in India until February 2009. The International Vaccine Institute, with funding from the Bill & Melinda Gates Foundation, aims at introducing vaccines against these two enteric diseases into the public-health programmes of developing countries (6). In this paper, we sought to assess the factors influencing individual and community participations, by analyzing data from two consecutive vaccine trials through spatial and multi-level techniques.

## MATERIALS AND METHODS

### Study setting and sample

The study was conducted in urban slum communities in Kolkata, capital of the state of West Bengal. Kolkata, the third largest city in India, has 14 million inhabitants living within an area of 1,450 sq km, making it one of the world's most densely-populated cities. The Kolkata Municipal Corporation consists of 141 civic administrative units called ward, with each ward having an office responsible for public health supervised by a medical officer. The study site comprises two contiguous wards (29 and 30) and encompasses an area of 0.99 sq km. Residents live in homes tightly-spaced together along winding sewage-littered pathways, and they rely on shared toilets and drinking-water (7). Seven referral government hospitals, various traditional practitioners, private medical clinics of qualified and unqualified physicians, and private nursing homes provide healthcare in Kolkata. Five health clinics (Fig.) were set up in the study area for primary care, and cases were referred to the Infectious Diseases Hospital (IDH), which is within a few km from the study site, and B.C. Roy Children Hospital, which is adjacent to the study site.

**Fig.**
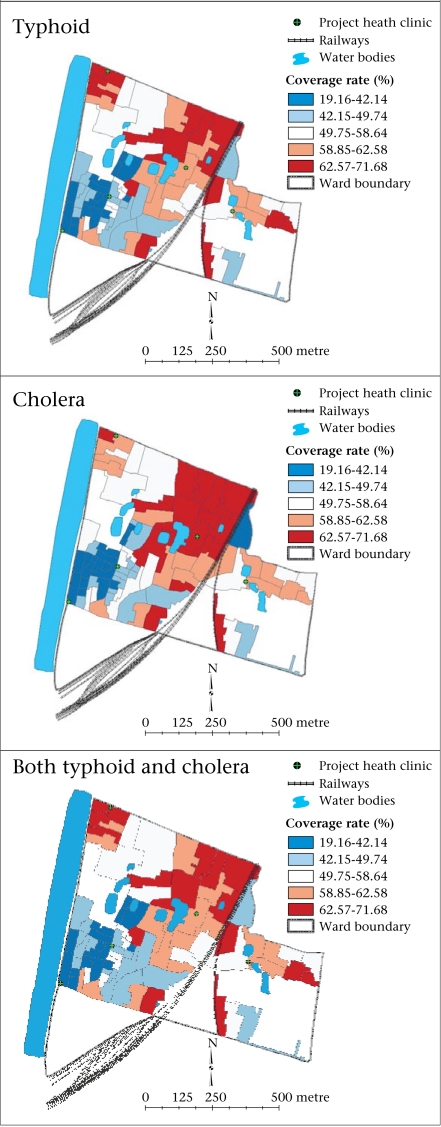
Local Bayes smoothing rate (%) of participation in typhoid vaccine, cholera vaccine, and both trials shown in quintile distribution of rates, Kolkata, India

The project staff conducted a baseline census in early 2002 to enumerate the people of the study area, which was followed by a second census one year later. Information on individual-level characteristics, such as age, gender, level of education, and socioeconomic status of the household, was collected during the census surveys. For the cholera vaccine trial, the study area was expanded to include residents of the southern part of Ward 29 and the whole of Ward 33. There were about 67,400 people living in the common trial area during the time of vaccination. However, for the purposes of this study, this additional area was not included in the analysis.

### Typhoid and cholera vaccination trials

The characteristics of the two vaccine trials are shown in Table 1. The new-generation Vi polysaccharide typhoid fever vaccine (Typherix) was used in the typhoid vaccine trial and was donated by GlaxoSmithKline. This vaccine is safe, requires single dosing, has consistent efficacy (64-77%), and has less strict cold-chain requirements, making the vaccine more adaptable for use in public-health settings in developing countries (8–10). Eligible participants for the typhoid vaccine trial were healthy, afebrile, non-pregnant, and non-lactating residents who were aged two years and older. The study subjects were randomized by geographical cluster to take either the Vi polysaccharide vaccine or the active control hepatitis A vaccine (Havrix) and were blinded. Eighty clusters were defined using the geographic information system (GIS), which defined clusters by connecting geographic features of households to give an average population (about 700) in each cluster. Vaccination was conducted from November to December 2004.

**Table 1.** Participation rate in typhoid and cholera vaccine trials among subjects aged two years and above, Kolkata, India

Participated in typhoid trial	Participated in cholera vaccine trial			Total (n=59,533)
	Yes (n=34,730)	No (n=20,210)	Not illegible* (n=4,593)	
Yes	28,099 (74.6)	9,581 (25.4)	0 (0.0)	37,680
No	5,994 (28.8)	10,262 (49.2)	4,593 (22.0)	20,849
Not illegible†	637 (63.4)	367 (36.6)	0 (0.0)	1,004

Figures in parentheses indicate percentages;

*Either pregnant during the vaccine campaign or migrated out before the cholera vaccine campaign;

†Febrile, pregnant, or lactating during the typhoid vaccine campaign

The cholera vaccine trial used a two-dose primary regimen of the oral killed whole-cell bivalent (*Vibrio cholerae* O1 and O139) cholera vaccine. This vaccine, recently licensed in India as *Shanchol* (Shantha Biotech, Hyderabad, India), was reformulated by scientists of the International Vaccine Institute to conform to the international and WHO standards. Like the typhoid vaccine trial, the design of the cholera vaccine trial was cluster-randomized and double-blinded, with the *Escherichia coli* K12 used as placebo. In the cholera vaccine trial, the unit of cluster was the premise, which is pre-assigned by the local municipal office. Each premise consists of one or more contiguous households (huts or if in a building, single rooms) sharing latrines and water sources. Healthy subjects aged one year and older residing in the study area and women who were not pregnant were eligible for participating in the cholera trial. Recruitment for the cholera trial was conducted from July to September 2006 with a two-week gap between the first and the second doses.

Non-participants in the study were defined as individuals who did not present in the vaccine trial centres for dosing. Participants in the two trials were blinded to the study agent that they received and were also unaware of the type of agent administered according to population cluster. Since our aim was to determine the factors associated with participation in the two consecutive vaccine trials, we used a common target population for both the trials by restricting the geographic area to the common study site where the typhoid trial was performed and the age-group, including only residents aged two years and older.

### Cluster and ecological variables

In this analysis, clusters were based on the typhoid vaccine trial using empirically-defined geographic units because the clusters (premises) in the cholera vaccine trial were very heterogenous with regard to population-size that may incorporate bias in cluster-level variations, We then used the empirically-divided 80 geographic units for conducting multi-level analysis assuming that social contacts to be strong within such smaller geographic units. Individual-level data on vaccination and socioeconomic status were aggregated for those 80 clusters to obtain ecological data for conducting the multi-level analysis.

### Empirical Bayes map

We used local Empirical Bayes estimation to map the spatial patterns of the vaccine coverage. The coverage was computed as a weighted sum of the rate observed at that location, and a prior mean was based on the first order of neighbours. The estimated coverage of each cluster shrunk towards the mean of the first order of neighbours (11).

### Statistical analysis

Multi-level models were used for analyzing the hierarchical structured data in which the individuals were clustered within the cluster (communities). This method enables investigation of the effects of variables measured at different levels (12,13), thus predicting the participation of an individual in the typhoid vaccine and cholera vaccine trials, and in the two trials adjusting for the community-level variation of participation in the trials. Unlike estimating fixed effects only in a regression model, the multi-level model offers estimation of both fixed and random effects (14); thus, variations in the community level can be known and adjusted through employing the model. Since the diverse community characteristics were observed in the study area, we assumed considerable variations in vaccine uptake among communities, which led us to choose the multi-level model for predicting the factors relating to participation in the two trials. We used HLM for Windows version (version 6.02a) (Scientific Software International, Inc., Lincolnwood, USA) for the multi-level (hierarchical) analysis.

### Ethics

The ethics committee of the National Institute of Cholera & Enteric Diseases, the Health Ministry Screening Committee of India, and the International Vaccine Institute Institutional Review Board approved the study protocol.

## RESULTS

Of the 59,533 individuals in the common target population of the two trials, 1,004 and 4,593 were ineligible, respectively, for the typhoid vaccine and cholera vaccine trials; 54,940 individuals were, thus, included in this analysis. After excluding ineligible subjects, participation was 64% and 63% in the typhoid vaccine and cholera vaccine trials respectively. Table 1 shows the participation of eligible subjects in either trial. Moreover, those who participated in the typhoid vaccine trial were more likely to participate in the subsequent cholera vaccine trial [odds ratio: 5.02; 95% confidence interval (CI) 4.83-5.22] compared to those who did not participate in the typhoid vaccine trial. The individual, household and cluster-level characteristics of the participants and non-participants for the two trials are shown in Table 2.

**Table 2.** Characteristics of participants and non-participants in typhoid and cholera vaccine trials

Characteristics	Participants		Non-participants	
	%	Mean	%	Mean
Typhoid (participants=37,680, non-participants=20,849)				
Age (years)		27.32		31.07
Male	52.55		59.12	
Hindu	61.47		51.40	
Distance (metre) from household to health outpost		158.22		148.12
Age (years) of household head		52.08		52.89
Male household head	79.28		80.62	
Educated household head*	35.43		38.34	
Household-size (no.)		7.65		7.84
Household owning motorbike	5.07		6.64	
% of Hindu in the cluster		61.67		50.95
Population age (years) in the cluster		28.83		28.22
% of males in the cluster		53.88		54.22
% of educated household heads in the cluster		36.71		35.69
% of households owning motorbike in the cluster		5.68		5.55
Cholera (participants=34,730, non-participants=20,210)				
Age (years)		27.04		30.39
Male	50.46		59.93	
Hindu	62.29		49.93	
Distance (metre) from household to health outpost		157.59		149.19
Age (years) of household head		51.98		52.28
Male household head	79.11		81.25	
Educated household head*	35.26		37.18	
Household-size (no.)		7.61		7.98
Household owning motorbike	4.88		6.87	
% of Hindu in the cluster		62.35		49.99
Population age (years) in the cluster		28.97		27.93
% of males in the cluster		53.77		54.33
% of educated household heads in the cluster		37.17		34.60
% of households owning motorbike in the cluster		5.71		5.44
Both typhoid and cholera (participants=28,099, non-participants=25,837)				
Age (years)		26.68		30.14
Male	50.58		59.62	
Hindu	64.60		50.73	
Distance (metre) from household to health outpost		160.22		148.76
Age (years) of household head		52.08		52.18
Male household head	78.72		81.30	
Educated household head*	35.39		36.99	
Household-size (no.)		7.54		7.93
Household owning motorbike	4.69		6.61	
% of Hindu in the cluster		64.81		50.58
Population age (years) in the cluster		29.14		28.04
% of males in the cluster		53.74		54.25
% of educated household heads in the cluster		37.56		34.90
% of households owning motorbike in the cluster		5.80		5.43

*Completed at least secondary education (10 years of schooling)

To predict the participation of an individual in the vaccine trials, the following three models were created: (a) no covariates were included (null model); (b) inclusion of a number of fixed individual-level terms; and (c) inclusion of a number of fixed individual- and cluster-level terms. The results of all the three models are shown in Table 3. The results of the unit-specified model with robust standard error are shown in terms of estimates and the ratios of the estimates to their standard errors (*t*-ratios). As a cut-off, if the ratio exceeds 2.0, the estimates are deemed significantly different from zero. Since the response variable was measured in logit scale, the estimates for the constants were 0.63, 0.58, and 0.10 for typhoid vaccine, cholera vaccine, and both the trials respectively; when untransformed it implies that the probability of participation in the typhoid vaccine, cholera vaccine, and in both the trials for all individuals across all clusters was 65%, 64%, and 52% respectively. The results also showed the substantial cluster-level differences (*t*-ratio=11.1) in participation in the typhoid vaccine trial with a grand mean of around 0.63 and a variance of 0.25. The cluster-level differences in participation in the cholera vaccine trial was also substantial (*t*-ratio=12.6). However, the cluster-level difference in participation in both the trials was not substantial. Upon estimating the residuals, the worst and best clusters had residuals of -1.81 and 1.08 respectively for the typhoid vaccine trial; only 23% participated in the worst clusters and 85% participated in the best clusters. Similarly, the worst communities in the cholera vaccine trial achieved only 43% coverage, and the best communities achieved 81% coverage. The trend remained the same when looking at coverage in both the trials (18% in the worst communities and 73% in the best communities).

**Table 3.** Model estimates and ratio of estimate to standard error (in parenthesis)

Variable	Typhoid (n=58,529)			Cholera (n=54,940)			Typhoid and cholera (n=53,936)		
	Model 1	Model 2	Model 3	Model 1	Model 2	Model 3	Model 1	Model 2	Model 3
Fixed-term: individual level									
Constant	0.633 (11.1)	1.574 (7.8)	1.728 (7.5)	0.578 (12.6)	1.243 (8.7)	1.460 (8.2)	0.100 (1.9)	0.882 (4.9)	1.101 (4.9)
Age (years)		-0.013** (18.8)	-0.013** (18.8)		-0.014** (17.0)	-0.014** (16.9)		-0.015** (19.4)	-0.015** (19.3)
Male		-0.278** (9.2)	-0.276** (9.1)		-0.385** (11.8)	-0.382** (11.7)		-0.374** (11.1)	-0.372** (11.1)
Hindu		-0.060 (0.4)	-0.245 (1.2)		0.387** (3.0)	0.134 (0.7)		0.128 (0.8)	-0.135 (0.6)
Distance (metre) to health outpost from household		-0.000 (0.4)	-0.000 (0.7)		-0.000 (0.1)	-0.000 (0.7)		-0.000 (0.0)	-0.000 (0.5)
Age (years) of household head		-0.005** (3.3)	-0.005** (3.3)		-0.003* (2.2)	-0.003* (2.3)		-0.001 (1.3)	-0.002 (1.4)
Male household head		0.036 (0.8)	0.039 (0.8)		0.031 (0.6)	0.035 (0.6)		0.005 (0.1)	0.009 (0.1)
Educated household head		-0.198** (4.0)	-0.199** (4.1)		-0.215** (4.6)	-0.219** (4.7)		-0.191** (4.0)	-0.193** (4.0)
Household-size		0.006 (1.1)	0.007 (1.2)		0.000 (0.1)	0.001 (0.2)		-0.000 (0.0)	0.000 (0.0)
Household owning motorbike		-0.318** (4.1)	-0.321** (4.2)		-0.413** (6.8)	-0.417** (6.8)		-0.439** (6.7)	-0.444** (6.8)
Fixed term: community level									
% of Hindus in the community			0.013** (4.7)			0.004* (2.1)			0.010** (4.1)
Average age (years)			-0.045 (1.4)			0.027 (1.2)			-0.008 (0.3)
% of males in the community			-0.015* (2.1)			-0.014** (3.3)			-0.016** (2.9)
% of educated household heads in the community			0.002 (0.3)			0.003 (0.7)			0.003 (0.7)
% of households owning motorbike in the community			-0.000 (0.0)			-0.021 (1.8)			-0.008 (0.7)
Random terms: community level									
Constant	0.257	0.347	0.200	0.164	0.136	0.088	0.225	0.257	0.131
Intra-cluster correlation coefficient	0.204	0.257	0.166	0.141	0.119	0.081	0.184	0.204	0.116

The results are expressed as logits;

**p<0.01;

*p<0.05

Model 2 included a number of fixed individual-level terms and its estimates are in Table 3. In each of the trials, individuals with the following characteristics had higher participation than their counterparts: younger age, female, from a household with a non-educated household head, and low socioeconomic status. Participation by Hindus was higher than by Muslims in the cholera vaccine trial. Individuals with a younger household head had higher participation rates in the typhoid vaccine and cholera vaccine trials compared to individuals with an older household head. The slight reduction of the cluster-level variance in Model 2 for the cholera vaccine trial from Model 1 indicated the slight differences in the predictor variability between clusters. After controlling for the individual-level characteristics that vary from cluster to cluster, participation in the worst and best communities are now 37% and 93% respectively for the typhoid vaccine trial, 62% and 89% respectively for the cholera vaccine trial, and 30% and 85% respectively for both the trials, which indicates a narrower range compared to the initial estimate from Model 1.

In Model 3, we added the cluster-level terms in addition to the individual-level terms. The estimate for the constant is now the log-odds of participation in a trial for the ‘average’ individual living in an average religiously-distributed cluster, average age, average percentage of males, average percentage of educated households, and average percentage of households of high socioeconomic status. The estimates of the individual-level fixed effects remained stable, except for religion (Hindu) while the coefficient for the extra terms showed that individuals in a Hindu- or female-dominated community had higher participation rates in each trial and in both the trials. The inclusion of extra terms in Model 3 resulted in a marked reduction of cluster-level variance from Model 1. The variance is significantly different from zero, and the contrast between predicted levels for the ‘average’ individual from the best and the worst communities is now a difference between 51% and 93% for the typhoid vaccine trial, 66% and 90% for the cholera vaccine trial, and 44% and 86% for both the trials. The intra-cluster correlation coefficient from the models ranged from 8% to 16%, indicating a significant amount of cluster-level variation in participation in the trials even after controlling for the individual and ecological characteristics.

The local empirical Bayes map shows a strong regional pattern of participation in the trial with a higher rate of participation noted in the northern part compared to that in the southern part of the study area (Fig.).

## DISCUSSION

Our results illustrate that those who participated in the initial (typhoid) vaccine trial were more likely to participate in the subsequent (cholera) vaccine trial compared to those who did not participate in the initial trial, suggesting that the vaccine trials spaced out in an 18-month interval did not affect participation in a subsequent trial. One limitation of the study is that we were unable to locate and detect individuals included in the census but who migrated outside the area before the vaccine trials; however, the population is relatively stable with a <5% annual migration; thus, we believe that this will not substantially change the results of the analysis.

The participation in the two trials was mainly influenced by various individual- and community-level factors. These factors were mostly similar in both typhoid and cholera vaccine trials with only a few exceptions. The younger people were more likely to participate in both the trials than the older people. Typhoid fever is thought of as a disease of young children (15), and the incidence of cholera is higher in younger people (7), which could have encouraged the young people to participate in the trials. The negative association of participation with households owning a motorbike, which is an indicator of economic status, may suggest that poorer people consider the campaign as an opportunity to get free vaccines (15). Participation in the trials was inversely related to the literacy and economic status of household head. In contrast, younger parents were keen to vaccinate their children, which is promising. Compared to HIV vaccine trials (16), women were more likely to participate in vaccine trials against enteric diseases compared to men.

Although religion at the individual level did not play any significant role in participation in all the trials, residents of predominantly Hindu communities were more likely to participate in the trials compared to residents of predominantly Muslim communities. This may be due to some preconceived notions among Muslim communities relating to vaccines (2), which could perhaps result in lower participation by Muslims. The inclusion of community-level factors in the model resulted in narrower range between the worst and the best community participation, suggesting that, within the clusters, individuals may share common attitudes and beliefs that may be targeted for behavioural change interventions, leading to improved participation in vaccine trials and campaigns.

The success of a vaccine trial or a vaccination campaign depends on many factors, including mobilization of the community. The social and cultural factors may also influence how vaccinations are interpreted (3). The high-coverage areas in the maps suggest the strong perceptions and community behaviours towards prevention of diseases, thereby motivating people to get vaccinated (3,17). The maps also guide interpretation of community-level obstacles to participation in vaccine trials and campaigns, and together with the outcomes of community-level analysis, they may suggest which factors discourage people not to participate.

The trials included in this analysis delivered vaccines through massive mobilization and recruitment of individuals in a short period, which may be similar to vaccination campaigns. However, unlike real public-health conditions, both the trials obtained informed consent from the community and individuals. Such procedures may have influenced community behaviour in vaccine uptake. More recently, behavioural change and prevention models were developed to provide better understanding of participation and non-participation in vaccination programmes (18,19). These models explicitly articulate the presumed mechanisms by which the changes in behaviour are brought about and guide the development of behavioural change interventions accompanying vaccine campaigns. While various models (17,20) have been used for developing behavioural change interventions in vaccine campaigns (21), identification of factors that may impact the success of vaccination and identification of acceptable and convenient sites for vaccine delivery, respected sources for information about the vaccine, e.g. health clinic personnel and community health volunteers, should be considered for successful vaccination campaigns. The lack of application of behavioural principles to understanding and designing strategies to introduce and improve vaccination programmes has limited the success of vaccination programmes and levels of participation in numerous contexts (21). Further studies of community behaviour to enhance the understanding and design of strategies to introduce and improve vaccination programmes would be useful.

## ACKNOWLEDGEMENTS

Financial support was provided by the Bill & Melinda Gates Foundation through the Diseases of Most Impoverished Program and the Cholera Vaccine Initiative administered by the International Vaccine Institute (IVI), Seoul, Korea. Current donors providing unrestricted support to the IVI include the Republic of Korea, Sweden, and Kuwait. The authors are grateful for the contributions by staff members of the IVI and the National Institute of Cholera & Enteric Disease, Kolkata, India.
